# Complete mitochondrial genome of the Arctic rainbow smelt *Osmerus dentex* (Osmeriformes, Osmeridae)

**DOI:** 10.1080/23802359.2018.1501301

**Published:** 2018-08-27

**Authors:** Evgeniy S. Balakirev, Alexandra Yu. Kravchenko, Nikolai S. Romanov, Francisco J. Ayala

**Affiliations:** a Department of Ecology and Evolutionary Biology, University of California, Irvine, CA, U.S.A;; b National Scientific Center of Marine Biology, Far Eastern Branch, Russian Academy of Sciences, Vladivostok, Russia;; c School of Natural Sciences, Far Eastern Federal University, Vladivostok, Russia

**Keywords:** Arctic rainbow smelt *Osmerus dentex*, European smelt *O. eperlanus*, Atlantic rainbow smelt *O. mordax*, Osmeridae, Mallotus, Hypomesus, Osmerus, mitochondrial genome

## Abstract

The complete mitochondrial genome was sequenced in two individuals of the Arctic rainbow smelt *Osmerus dentex*. The genome sequences are 16,615 and 16,616 bp in size, and the gene arrangement, composition, and size are very similar to the other smelt mitochondrial genomes published previously. The difference between the two *O. dentex* genomes studied is 0.25%, that is, 5.0 times higher in comparison with close species, the European smelt *O. eperlanus* studied previously. The level of mitochondrial genome divergence between *O. dentex* and close osmerid fishes, *O. eperlanus*, and *O. mordax* is high enough (6.86–7.54%) to consider all of them as separate biological species.

The Arctic rainbow smelt *Osmerus dentex* Steindachner and Kner has wide distribution in the North Pacific regions from the Wonsan coastal area (North Korea) and the Sea of Okhotsk to Barkley Sound (British Columbia), northward to the Bering Sea, and the Arctic Ocean from the White Sea to the Chukotka Sea (eastern Siberia) (Kottelat and Freyhof 2007). Berg (1949) considered *O. dentex* as a subspecies of the European smelt *O. eperlanus* (L.). McAllister (1963) distinguished two subspecies, *O. eperlanus eperlanus* and *O. eperlanus mordax* sensu lato including *O. eperlanus dentex*. Kljukanov and McAllister (1973) concluded that *O. mordax* consists of two subspecies, *O. mordax mordax* and *O. mordax dentex* (https://www.ncbi.nlm.nih.gov/Taxonomy/Browser/wwwtax.cgi?id=8013).

Using the mitochondrial (mt) DNA restriction fragment length and cytochrome b sequence polymorphism, Taylor and Dodson (1994) revealed a high level of divergence (5.6–8.9%) between the Arctic, European, and Atlantic smelts suggesting the species status for each of them. The morphological data (Shedko 2001) presented new arguments to increase the *O. dentex* status from subspecies to species level. However, the scientific literature and particularly the GenBank Taxonomy Browser still list *O. dentex* as a subspecies of *O. mordax*.

To increase the power of the molecular taxonomy analysis of this complex fish group we have sequenced two complete mt genomes of *O. dendex* (GenBank accession numbers MH370836 and MH370837) from the Amur Bay of the Sea of Japan (43˚13′ 17,256″ N; 131˚55′ 37,113″ E). The primers were designed with the program mitoPrimer_V1 (Yang et al. 2011). The fish specimens are stored at the museum of the National Scientific Center of Marine Biology, Vladivostok, Russia (www.museumimb.ru) under accession numbers MIMB35006 and MIMB35007.

The *O. dentex* mt genome sequences are 16,615 and 16,616 bp in size and the gene arrangement, composition, and size are very similar to the smelt fish genomes published previously. We detected 41 single nucleotide and one length differences between the haplotypes 394Omd1 and 397Omd4; total sequence divergence (*D*_xy_) was 0.0025 ± 0.0004. The difference between the two *O. dentex* genomes studied is relatively high in comparison with close species, the European smelt *O. eperlanus*, which demonstrated 5.0 times smaller difference (0.0005 ± 0.0001) between two genomes studied previously (Balakirev et al. 2018), which corresponds with much wider species range in *O. dentex* than in *O. eperlanus* (McAllister 1984; Kottelat and Freyhof 2007), but it could also indicate the overfishing in *O. eperlanus* populations (Hutchinson and Mills 1987).

The comparison of mt genomes now obtained with other complete mt genomes of related groups available in GenBank including genera *Hypomesus*, *Osmerus*, and *Mallotus* reveals a close affinity of *O. dentex* to other *Osmerus* species (Figure 1). The level of sequence divergence between *O. dentex*, *O. eperlanus*, and *O. mordax* is still high enough (6.86–7.54%) to consider all of them as separate biological species. Similar values of interspecific divergence between osmerid fishes were reported previously (e.g. Skurikhina et al. 2013 and references therein).

**Figure 1.**
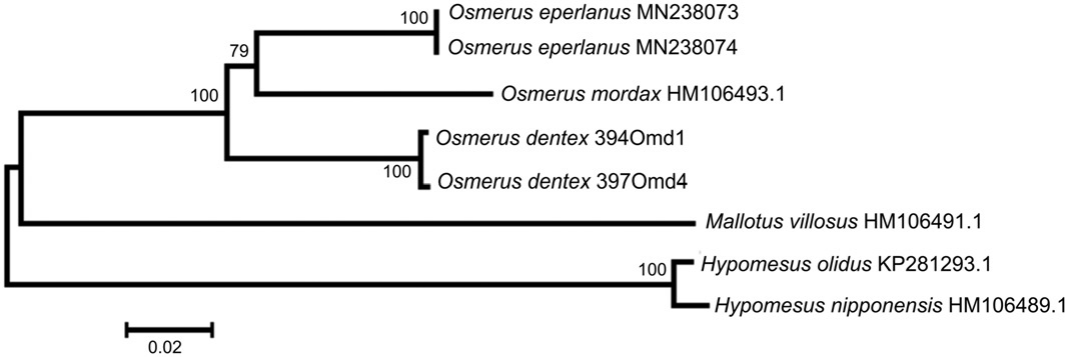
Maximum likelihood tree for the Arctic rainbow smelt *Osmerus dentex* specimens 394Omd1 and 397Omd4, and GenBank representatives of the order Osmeriformes. The tree is based on the General Time Reversible + gamma + invariant sites (GTR + G + I) model of nucleotide substitution. The numbers at the nodes are bootstrap percent probability values based on 1000 replications.
